# Causal relationship between dried fruit intake and frozen shoulder: Two-sample Mendelian randomization

**DOI:** 10.1097/MD.0000000000036099

**Published:** 2023-11-17

**Authors:** Guang-Hua Deng

**Affiliations:** a Ya’an City Hospital of Traditional Chinese Medicine, Ya'an, Sichuan, China.

**Keywords:** dried fruit, frozen shoulder, intake, Mendelian randomization

## Abstract

To investigate the causal relationship between dried fruit intake and frozen shoulder using Mendelian randomization (MR). Genome wide association studies were conducted to pool data and select genetic loci independently associated with dried fruit intake and frozen shoulder in people of European ancestry as instrumental variables. Three MR analyses, inverse variance weighting, weighted median and MR-Egger, were used to investigate the causal relationship between dried fruit intake and frozen shoulder. Heterogeneity and multiplicity tests were used, and sensitivity analyses were conducted using the leave-one-out method to explore the robustness of the results. The inverse variance weighting results showed an OR (95 % CI) of 0.52 (0.34–0.80), *P* = .003, suggesting that there is a causal relationship between dried fruit intake and frozen shoulder. And no heterogeneity and multiplicity were found by the test and sensitivity analysis also showed robust results. The present study used a two-sample MR analysis, and by analyzing and exploring the genetic data, the study showed that too little intake of dry fruits is a risk factor for developing frozen shoulder.

## 1. Introduction

Frozen shoulder, also known as adhesive capsulitis,^[1]^ is a pathological condition characterized by pain and limitation of joint motion in the shoulder.^[2]^ There are usually no significant findings in the patient’s history, clinical examination, or imaging assessment to explain the loss of motion or pain.^[3,4]^ In recent years, many studies have found that dried fruit intake is associated with the development of several diseases, such as excessive low intake of dried fruits increases cardiovascular disease,^[5]^ and moderate intake of dried fruits is a protective factor for asthma^[6]^ and low back pain.^[7]^ However, studies on dried fruit intake and frozen shoulder are more lacking and the causal relationship is uncertain. The causal relationship between dried fruit intake and frozen shoulder still needs further investigation.

Mendelian randomization (MR), a genetic epidemiological method, is a useful tool to assess the causal role of dried fruit intake and frozen shoulder.^[8]^ By using genetic variants such as single nucleotide polymorphism (SNP) as instrumental variants that can modify disease risk factors or exposures, MR studies can enhance causal inference of exposure-outcome associations.^[9]^ According to Mendel laws of inheritance, genetic variants are not susceptible to confounding factors because they are randomly assigned during gamete formation.^[10]^ In addition, confounders and reverse causation can be minimized as genotypes cannot change as the disease progresses.^[11]^

To this end, we conducted a two-sample MR study to examine the association of dried fruit intake with causality in frozen shoulder. We aimed to provide significant evidence for the causal role of dried fruit intake in causing frozen shoulder.

## 2. Information and methods

### 2.1. Data sources

Genome-wide association study (GWAS) data on dried fruit intake and frozen shoulder were obtained from the IEU OpenGWAS project (mr cieu.ac.uk) website. The website was accessed on June 06, 2023, and all data ultimately obtained for this study were from a European population. These included dried fruit intake (ukb-b-16576) with 9,851,867 SNPs and a sample size of 421,764 individuals, and frozen shoulder (ebi-a-GCST90000512) with 15,184,371 SNPs and a sample size of 451,099 individuals. This study was a reanalysis of previously collected and published publicly available data and therefore did not require additional ethical approval.

### 2.2. Condition setting for SNPs as instrumental variables

The instrumental variable was highly correlated with exposure, with *F* > 10 as a strong correlation criterion.^[12]^ The instrumental variable is not directly related to the outcome, but only affects the outcome through exposure, that is, there is no genetic pleiotropy. In this study, the intercept term of the MR-Egger regression model was nonzero (*P* < .05), indicating the absence of genetic pleiotropy.^[13]^ Instrumental variables were not associated with untested confounding.^[14]^ The human genotype–phenotype association database Phenoscanner V2 was searched for phenotypes associated with the instrumental variables at the genome-wide significance level to determine whether these SNPs were associated with potential risk factors.^[15]^

### 2.3. SNP screening

Significant SNPs were screened from the GWAS pooled data of dried fruit intake (*P* < 5 × 10^−8^ was used as the screening condition)^[16]^; the linkage disequilibrium coefficient r^2^ was set to be 0.001 and the width of the linkage disequilibrium region to be 10,000 kb to ensure that the individual SNPs were independent of each other.^[17]^ The SNPs related to dried fruit intake screened above were extracted from the GWAS pooled data of frozen shoulder, while SNPs directly related to outcome indicators were excluded (*P* < 5 × 10^−8^). The *F*-value of each SNP was calculated, and SNPs with weak instrumental variables (*F*-value <10) were excluded.^[18]^ And the human genotype–phenotype association database was queried to screen for potentially relevant risk factor SNPs and exclude them.^[19]^

### 2.4. Methods of causality verification

The causal relationship between exposure (dried fruit intake) and outcome (frozen shoulder) was verified using mainly inverse variance weighted (IVW) as, supplemented by MR-Egger and Weighted median (WME) MR analyses with SNP as instrumental variables.

### 2.5. Sensitivity analysis

Sensitivity analyses were conducted using several methods. First, the Cochran Q test was used to assess the heterogeneity between the individual SNP estimates, and a statistically significant Cochran Q test proved that the analyses were significantly heterogeneous. Second, MR pleiotropy residual sum and outlier (MR PRESSO) was used to validate the results in the IVW model, to correct for the effect of outliers, and if outliers existed, they were removed and the analysis was repeated. Third, the horizontal multiplicity of SNPs was tested using the MR Egger intercept test (MR Egger intercept test), and if the intercept term in the MR Egger intercept test analysis was statistically significant, it indicated that the MR analysis had significant horizontal multiplicity. Fourth, “leave-one-out” sensitivity analyses were performed by removing a single SNP at a time to assess whether the variant drove the association between the exposure and outcome variables. Fifth, funnel plots and forest plots were constructed to visualize the results of the sensitivity analyses. *P* < .05 suggests that there is a potential causal relationship in the MR analyses, which is statistically significant. All statistical analyses were performed using the “TwoSampleMR” package in R software version 4.3.0.

## 3. Results

### 3.1. Instrumental variables

Forty-three SNPs that were strongly associated with dried fruit intake (*P* < 5 × 10^−8^) without chain disequilibrium (r^2^ < 0.001, kb = 10,000) were screened in the current study. Forty-three SNPs were left by taking the intersection with SNPs in the pooled data of the GWAS for frozen shoulder, and also by eliminating SNPs that were directly associated with the outcome metrics. In our study, the *F*-value of each SNP was >10, indicating the absence of weak instrumental variables (see Table 1 for details). We searched the human genotype–phenotype association database and found no potentially relevant risk factor SNPs.

**Table 1:** Information on the final screening of dried fruit intake SNPs from GWAS data (n = 43).

ID	SNP	Effect_Allele	Other_Allele	β	SE	*P*	*F*
1	rs10026792	A	G	0.0108465	0.0018423	3.90E−09	34
2	rs10129747	G	A	0.00935902	0.00168126	2.60E−08	30
3	rs10740991	C	G	0.016739	0.00185722	2.00E−19	81
4	rs10896126	G	A	−0.015009	0.00181919	1.60E−16	68
5	rs11037497	C	G	0.0104396	0.00168442	5.70E−10	38
6	rs11152349	A	G	0.00991215	0.0018176	4.90E−08	29
7	rs11586016	C	G	0.00987818	0.00173034	1.10E−08	32
8	rs11632215	C	A	−0.0141434	0.00258382	4.40E−08	29
9	rs11720884	G	A	0.0111797	0.00193554	7.60E−09	33
10	rs11772627	C	G	0.0183338	0.00217099	3.00E−17	71
11	rs11811826	A	T	0.0132178	0.0020058	4.40E−11	43
12	rs12137234	T	C	0.0102051	0.00183744	2.80E−08	30
13	rs1582322	G	A	0.00994346	0.00171571	6.80E−09	33
14	rs1622515	G	A	0.00991719	0.00167077	2.90E−09	35
15	rs1648404	T	C	0.00941595	0.00167357	1.80E−08	31
16	rs17175518	A	C	0.0114962	0.00197514	5.90E−09	33
17	rs17184707	T	C	−0.0114381	0.00204011	2.10E−08	31
18	rs1797235	C	G	−0.0100206	0.00174235	8.90E−09	33
19	rs2328887	C	T	0.0189482	0.00277607	8.80E−12	46
20	rs2533273	A	C	−0.00987659	0.00167714	3.90E−09	34
21	rs261809	G	A	−0.00962858	0.00167902	9.80E−09	32
22	rs3101339	C	A	0.0142595	0.00170546	6.20E−17	69
23	rs34162196	T	C	−0.0223628	0.00277168	7.10E−16	65
24	rs3764002	T	C	0.0131215	0.00190081	5.10E−12	47
25	rs4140799	A	G	0.0094566	0.00167846	1.80E−08	31
26	rs4149513	A	G	0.01173	0.00167139	2.20E−12	49
27	rs4269101	G	T	−0.0138099	0.00185921	1.10E−13	55
28	rs429358	C	T	0.0199445	0.00231362	6.70E−18	74
29	rs4800488	A	C	0.0119836	0.00167202	7.70E−13	51
30	rs57499472	C	T	0.00991231	0.00171869	8.10E−09	33
31	rs62084586	C	T	0.0133946	0.00226174	3.20E−09	35
32	rs72720396	G	A	0.0114265	0.00198541	8.70E−09	33
33	rs746868	G	C	−0.0129055	0.00171452	5.20E−14	56
34	rs75641275	C	A	−0.0141613	0.00238518	2.90E−09	35
35	rs7582086	T	G	−0.00962821	0.00167381	8.80E−09	33
36	rs7599488	T	C	−0.0104154	0.00168729	6.70E−10	38
37	rs7808471	C	T	−0.0115362	0.00178604	1.10E−10	41
38	rs7829800	G	A	−0.0104463	0.00178709	5.10E−09	34
39	rs7916868	T	A	0.00960741	0.00167167	9.10E−09	33
40	rs8081370	T	C	−0.0166666	0.00293795	1.40E−08	32
41	rs862227	G	A	−0.0091646	0.00167238	4.30E−08	30
42	rs893856	A	G	−0.013361	0.00234923	1.30E−08	32
43	rs9385269	T	C	0.0120673	0.00168183	7.20E−13	51

### 3.2. Causal relationship between dried fruit intake and frozen shoulder

**Figure 1.**
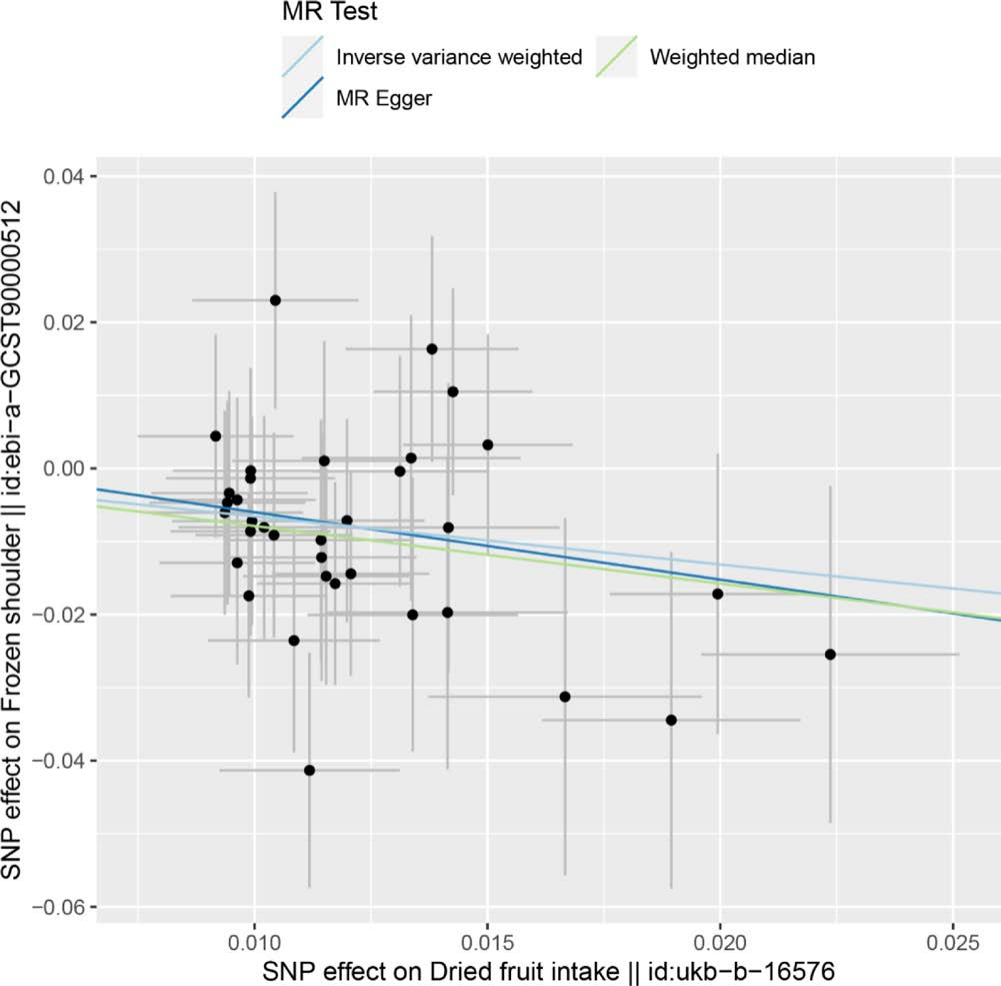
Scatter plot of dried fruit intake and frozen shoulder.

**Figure 2.**
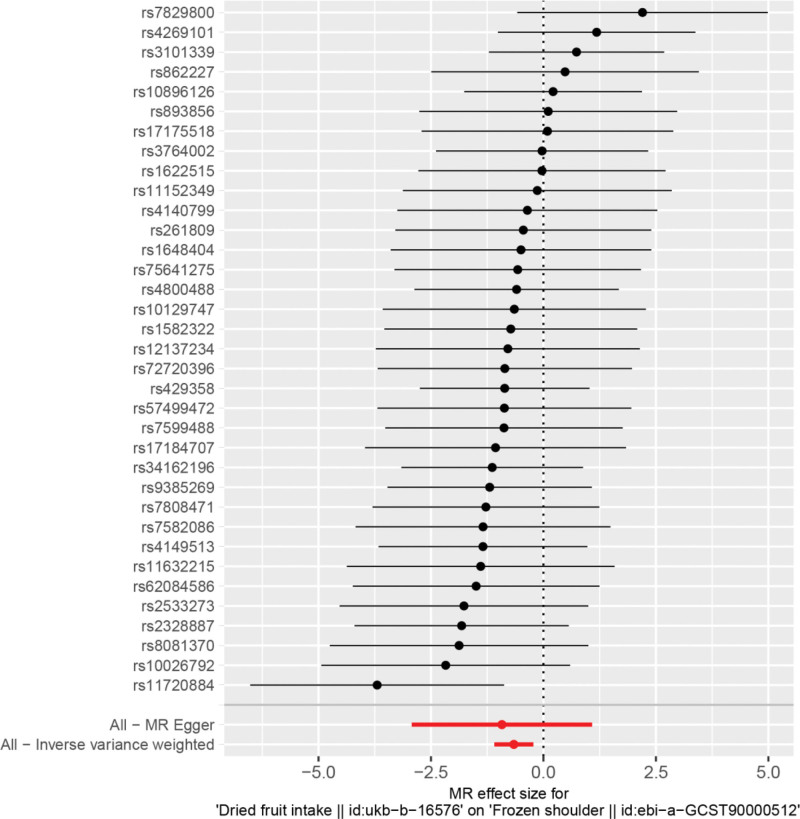
Forest plot of dried fruit intake and frozen shoulder.

By MR analysis, the results of both IVW and WME showed that there was a causal relationship between dried fruit intake and frozen shoulder. IVW:OR = 0.52, 95% CI = 0.34–0.80, *P* = .003; WME:OR = 0.45, 95% CI = 0.25–0.83, *P* = .010; (see Table 2 for details). We can see from both the scatter plot (Fig. 1) and the forest plot (Fig. 2) that dried fruit intake increases the risk of developing frozen shoulder.

**Table 2:** MR regression results of the 3 methods.

Method	β	SE	OR (95%CI)	*P*
IVW	−0.657	0.211	0.52 (0.34–0.80)	.003
WME	−0.788	0.307	0.45 (0.25–0.83)	.010
MR-Egger	−0.923	1.022	0.40 (0.05–2.95)	.373

### 3.3. Sensitivity analysis

**Figure 3.**
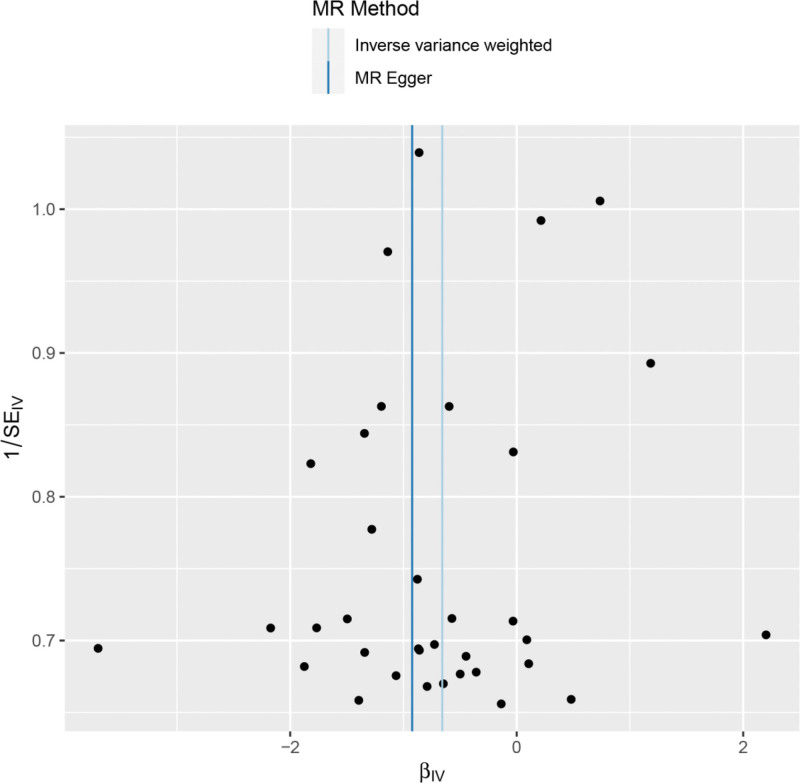
Funnel plot of dried fruit intake and frozen shoulder.

**Figure 4.**
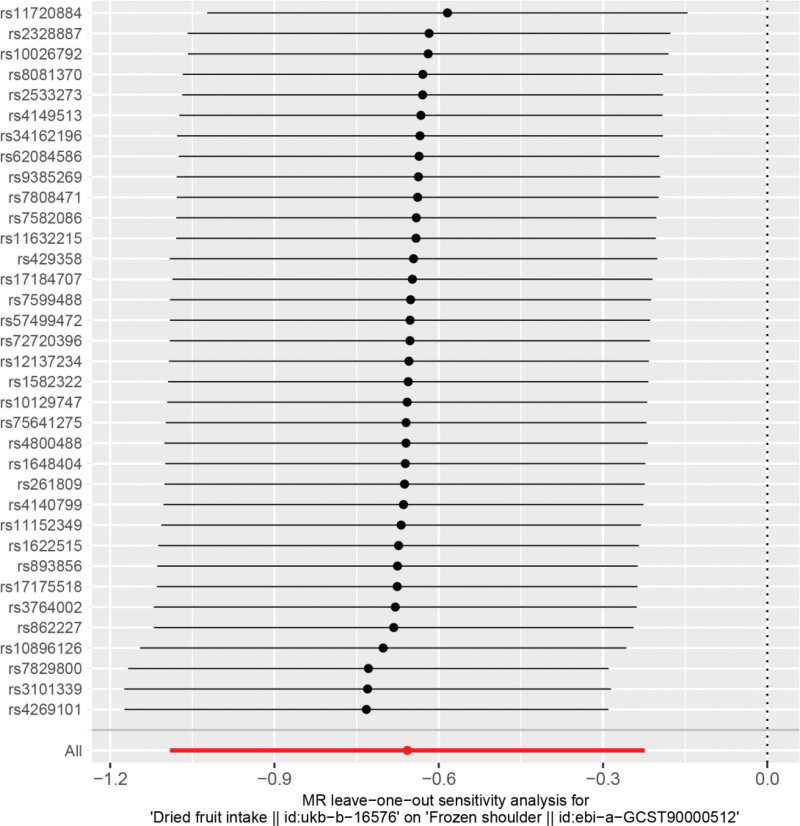
Analysis of dried fruit intake and frozen shoulder by the leave-one-out method.

Heterogeneity was tested using the IVW method (Cochran Q test, *P* = .959) and the results suggested that there was no heterogeneity. A funnel plot was drawn to show the heterogeneity results, as shown in Figure 3. MR-PRESSO was used to screen for SNPs that could lead to heterogeneity, and the results did not reveal any SNPs that would lead to heterogeneity in the results. The Global test results by MR-PRESSO suggested that there was no pleiotropy (*P* = .792). The “leave-one-out” method uses the IVW method by default, and as can be seen in Figure 4, no single SNP will have a large impact on the overall results after eliminating any SNP, indicating that the results are robust.

## 4. Discussion

It is known that excessively low intake of dried fruits may be an observational risk factor for frozen shoulder, but the causality of this association is unclear. Our MR study aimed to reveal the causal relationship between dried fruit intake and frozen shoulder. The two-sample MR results showed a causal association between dried fruit intake and frozen shoulder, with an OR (95 % CI) of 0.52 (0.34–0.80), *P* = .003, indicating that people with a low intake of dried fruits are at a higher risk of developing frozen shoulder compared to the general population.

The term “frozen shoulder” is often incorrectly used^[20,21]^ and attributed to other shoulder limitations such as rotator cuff tears or osteoarthritis. Subacromial lesions (e.g., rotator cuff tendinopathy, subacromial bursitis, and impingement syndrome) may also closely resemble frozen shoulder in the early stages.^[22]^ For appropriate management, it is important for the physician to establish the diagnosis. Frozen shoulder is estimated to affect 2% to 5% of the general population,^[23]^ and can be significantly painful and disabling. It most often affects people between the ages of forty and sixty and occurs more often in women than in men.^[24,25]^ Therefore, it is necessary and urgent to identify risk factors and develop effective public strategies to prevent frozen shoulder. Adverse lifestyle behaviors are clear risk factors for frozen shoulder.^[26]^ Previous studies have reported that too little intake of dried fruits is a risk factor for many diseases such as cardiovascular diseases, croup and lumbar. Similarly, the present study confirmed that dry fruit intake was negatively associated with the risk of frozen shoulder from a genetic point of view. In addition statistical evidence from sensitivity analyses strongly supports our findings. Therefore, awareness of the dangers of low intake of dried fruits should be increased. Screening for frozen shoulder in people with low intake of dried fruits should be increased, so that patients with frozen shoulder can be detected early and treated in time, which is beneficial to the prognosis of the patients. Increased intake of dried fruits should be promoted for patients with frozen shoulder.

The mechanism underlying the causal relationship between dried fruit intake and low back pain is unclear. Dried fruits are obtained from fresh fruits by using various drying techniques. They are important healthy snacks and are a rich source of dietary fiber, minerals, vitamins, and various bioactive compounds such as flavonoids and carotenoids.^[27]^ Dried fruits exert a variety of biological effects, including antioxidant, anti-inflammatory, anti-atherosclerotic, and anticancer effects.^[28,29]^ Experimental studies have shown that dried fruit intake inhibits pro-inflammatory cytokines and promotes the function of the musculoskeletal system.^[30,31]^ In clinical studies, many authors have found that daily intake of dried fruits has a protective effect on musculoskeletal health in both men and women.^[32,33]^ However, further experimental and clinical studies are needed due to limited evidence on the underlying mechanisms.

There are also some limitations of this study. Firstly, as all data are from people of European origin, the results are not representative of a truly randomized population sample and are not applicable to other so races. Second, although various sensitivity analyses have been performed in this study to test the hypotheses of the MR study, it is also difficult to completely rule out horizontal pleiotropy of instrumental variables. Finally, the current sample size of GWAS data is still not large enough, and more in-depth studies using more GWAS data are needed in the future.

## 5. Conclusion

The present study used a two-sample MR analysis, and by analyzing and exploring the genetic data, the study showed that too little intake of dry fruits is a risk factor for developing frozen shoulder.

## Author contributions

**Conceptualization:** Guang-Hua Deng.

**Data curation:** Guang-Hua Deng.

**Formal analysis:** Guang-Hua Deng.

**Funding acquisition:** Guang-Hua Deng.

**Investigation:** Guang-Hua Deng.

**Methodology:** Guang-Hua Deng.

**Project administration:** Guang-Hua Deng.

**Resources:** Guang-Hua Deng.

**Software:** Guang-Hua Deng.

**Supervision:** Guang-Hua Deng.

**Validation:** Guang-Hua Deng.

**Visualization:** Guang-Hua Deng.

**Writing – original draft:** Guang-Hua Deng.

**Writing – review & editing:** Guang-Hua Deng.
